# Supplementary material for: Prevalence of Human Papilloma Virus Infection in Bladder Cancer: A Systematic Review
Source: Diagnostics (Basel). 2022 Jul 20;12(7):1759. doi: 10.3390/diagnostics12071759 (PMC9318826; doi:10.3390/diagnostics12071759)
Supplement: Supplementary file 1 [file diagnostics-12-01759-s001.zip › diagnostics-1775896-supplementary.pdf]

Table S1a: Characteristics of the included studies ordered by study design: a) case-control;

First author	Year	Title	Type of study	Multicentre / Single Centre	Country/ies	Study period
Abdollahzadeh P, et al	2017	Association Between Human Papillomavirus and Transitional Cell Carcinoma of the Bladder	Case/control study	Single	Iran	2008-2011
Badawi H, et al	2008	Role of human papillomavirus types 16, 18, and 52 in recurrent cystitis and urinary bladder cancer among Egyptian patients	Case/control study	Single	Egypt	2001-2006
Barghi MR, et al	2005	Correlation between human papillomavirus infection and bladder transitional cell carcinoma	Case/control study	Single	Iran	199-2002
Gould VE, et al	2010	Human papillomavirus and p16 expression in inverted papillomas of the urinary bladder	Case/control study	Single	USA	-
Kim SH, et al	2014	Detection of human papillomavirus infection and p16 immunohistochemistry expression in bladder cancer with squamous differentiation	Case/control study	Single	Korea	2001-2011
Mete UK, et al	2018	Human Papillomavirus in Urothelial Carcinoma of Bladder: An Indian study	Case/control study	Single	India	-
Sarier M, et al	2019	Is There any Association between Urothelial Carcinoma of the Bladder and Human Papillomavirus? A Case-Control Study	Case/control study	Single	Turkey	Jan-Dec 2018
Shaker OG, et al	2013	Is there a correlation between HPV and urinary bladder carcinoma?	Case/control study	Single	Egypt	-
Shigehara K, et al	2011	Etiologic role of human papillomavirus infection in bladder carcinoma	Case/control study	Single	Japan	1997-2009
Steinestel J, et al	2013	Overexpression of p16INK4a in Urothelial Carcinoma In Situ Is a Marker for MAPK-Mediated Epithelial-Mesenchymal Transition but Is Not Related to Human Papillomavirus Infection	Case/control study	Single	Germany	2001-2011
Tekin MI, et al	1999	Human papillomavirus associated with bladder carcinoma? Analysis by polymerase chain reaction	Case/control study	Single	Turkey	-

Table S1b: Characteristics of the included studies ordered by study design: b) cross-sectional design.

First author	Year	Title	Type of study	Multicentre / Single Centre	Country/ies	Study period
Aggarwal S, et al	2009	Koilocytosis: correlations with high-risk HPV and its comparison on tissue sections and cytology, urothelial carcinoma	Retrospective observational study	Single	India	-
Alexander RE, et al	2012	p16 expression is not associated with human papillomavirus in urinary bladder squamous cell carcinoma	Retrospective observational study	Single	USA	1992-2011
Alexander RE, et al	2013	Human papillomavirus is not an etiologic agent of urothelial inverted papillomas	Retrospective observational study	Multi	USA<br>Spain<br>Italy<br>France	1985-2005
Alexander RE, et al	2014	The expression patterns of p53 and p16 and an analysis of a possible role of HPV in primary adenocarcinoma of the urinary bladder	Retrospective observational study	Multi	USA<br>Spain<br>Italy<br>France	-
Ben Selma W, et al	2010	Investigation of human papillomavirus in bladder cancer in a series of Tunisian patients	Observational study	Single	Tunisia	2003-2004
Berrada N, et al	2013	Human papillomavirus detection in Moroccan patients with bladder cancer	Prospective study	Single	Morocco	-
Chan KW, et al	1997	Prevalence of six types of human papillomavirus in inverted papilloma and papillary transitional cell carcinoma of the bladder: an evaluation by polymerase chain reaction	Retrospective observational study	Single	China	1987-1994
Chapman-Fredricks JR, et al	2013	High-risk human papillomavirus DNA detected in primary squamous cell carcinoma of urinary bladder	Retrospective observational study	Single	USA	-
Collins K, et al	2020	Prevalence of high-risk human papillomavirus in primary squamous cell carcinoma of urinary bladder	Retrospective observational study	Single	Texas	2009-2019

Cooper K, et al	1997	Human papillomavirus and schistosomiasis associated bladder cancer	Retrospective observational study	Single	South Africa	-
De Gaetani C, et al	1999	Detection of human papillomavirus DNA in urinary bladder carcinoma by in situ hybridisation	Retrospective observational study	Single	Italy	1995-1997
Fioriti D, et al	2003	Urothelial bladder carcinoma and viral infections: different association with human polyomaviruses and papillomaviruses	Comparative study	Single	Italy	-
Gazzaniga P, et al	1998	Prevalence of papillomavirus, Epstein-Barr virus, cytomegalovirus, and herpes simplex virus type 2 in urinary bladder cancer	Retrospective observational study	Single	Italy	-
Golovina DA, et al	2016	Loss of Cell Differentiation in HPV-Associated Bladder Cancer	Retrospective observational study	Single	Russia	-
Gopalkrishna V, et al	1995	Detection of human papillomavirus DNA sequences in cancer of the urinary bladder by in situ hybridisation and polymerase chain reaction	Retrospective observational study	Single	India	-
Javanmard B, et al	2019	Human Papilloma Virus DNA in Tumor Tissue and Urine in Different Stage of Bladder Cancer	Retrospective observational study	Single	Iran	2014-2016
Kamel D, et al	1995	Human papillomavirus DNA and abnormal p53 expression in carcinoma of the urinary bladder	Retrospective observational study	Single	Finland	1987-1992
Kim KH, et al	1995	Analysis of p53 tumor suppressor gene mutations and human papillomavirus infection in human bladder cancers	Retrospective observational study	Single	Korea	-
LaRue H, et al	1995	Human papillomavirus in transitional cell carcinoma of the urinary bladder	Retrospective observational study	Single	Canada	-
Llewellyn MA, et al	2018	Defining the frequency of human papillomavirus and polyomavirus infection in urothelial bladder tumours	Retrospective observational study	Single	UK	2005-2011

Lopez-Beltran A, et al	1996a	Human papillomavirus DNA as a factor determining the survival of bladder cancer patients	Retrospective observational study	Single	Spain	-
López-Beltrán A, et al	1996b	Human papillomavirus infection and transitional cell carcinoma of the bladder: Immunohistochemistry and in situ hybridization	Observational study	Single	Spain	-
Moghadam SO, et al	2020	Association of human papilloma virus (HPV) infection with oncological outcomes in urothelial bladder cancer	Prospective study	Single	Iran	-
Musangile FY, et al	2021	Detection of HPV infection in urothelial carcinoma using RNAscope: Clinicopathological characterization	Retrospective observational study	Single	Japan	2013-2019
Pichler R, et al	2015	Low prevalence of HPV detection and genotyping in non-muscle invasive bladder cancer using single-step PCR followed by reverse line blot	Prospective study	Single	Austria	-
Samarska IV, et al	2019	Condyloma Acuminatum of Urinary Bladder: Relation to Squamous Cell Carcinoma	Observational study	Single	*	-
Schmid SC, et al	2015	Human papilloma virus is not detectable in samples of urothelial bladder cancer in a central European population: a prospective translational study	Prospective study	Single	Germany	-
Shigehara K, et al	2013	Etiological correlation of human papillomavirus infection in the development of female bladder tumor	Prospective study	Single	Japan	1996-2010
Simoneau M, et al	1999	Low frequency of human papillomavirus infection in initial papillary bladder tumors	Retrospective observational study	Single	Canada	1990-1992
Tenti P, et al	1996	p53 overexpression and human papillomavirus infection in transitional cell carcinoma of the urinary bladder: correlation with histological parameters	Retrospective observational study	Single	Italy	-

Westenend PJ, et al	2001	Human papillomaviruses 6/11, 16/18 and 31/33/51 are not associated with squamous cell carcinoma of the urinary bladder	Retrospective observational study	Single	Netherlands	-
Yan Y, et al	2021	Human Papillomavirus Prevalence and Integration Status in Tissue Samples of Bladder Cancer in the Chinese Population	Retrospective observational study	Single	China	2015-2019
Yavuzer D, et al	2011	Role of human papillomavirus in the development of urothelial carcinoma	Retrospective observational study	Single	Turkey	-
Youshya S, et al	2005	Does human papillomavirus play a role in the development of bladder transitional cell carcinoma? A comparison of PCR and immunohistochemical analysis	Retrospective observational study	Single	England	-

Table S2. Demographics characteristics of studies enrolled.

First author	Sample size	Cases	Controls	Gender (by cases or total)		Gender (by controls)		Mean/median (SD/range) age by cases or total	Specimen type	
				Females	Males	Females	Males		Fresh	FFPE
Abdollahzadeh P; 2017	97	67	30	9	58	13	17	-	-	97
Aggarwal S; 2009	33	-	-	7	26	-	-	56.5 (28-80)	-	33
Alexander RE; 2012	69	-	-	21	48	-	-	69.5 (37-101)	-	69
Alexander RE; 2013	27	-	-	2	23	-	-	61 (32-78)	-	27
Alexander RE; 2014	36	-	-	-	-	-	-	61 (32-87)	-	36
Badawi H; 2008	80	60	20	13	47	-	-	49.37 (13.61/16-75)	60	-
Barghi MR; 2005	79	59	20	9	50	4	16	67 (10.8)	-	79
Ben Selma W; 2010	125	-	-	16	109	-	-	70** (28-99)	-	125
Berrada N; 2013	48	-	-	38	4	-	-	65 (32-86)	-	48
Chan KW; 1997	30	-	-	-	-	-	-	-	-	34
Chapman-Fredricks JR; 2013	14	-	-	6	8	-	-	-	-	14
Collins K; 2020	33	-	-	17	16	-	-	64 (59-91)	-	33
Cooper K; 1997	25	-	-	7	18	-	-	47** (29-72)	-	25
De Gaetani C; 1999	43	-	-	5	38	-	-	66.3 (19.8/36-85)	-	-
Fioriti D; 2003	52	32	20	4	28	7	13	64.6 (20-92)	52	-
Gazzaniga P; 1998	45	35	10	6	29	-	-	-	35	-
Golovina DA; 2016	101	-	-	17	84	-	-	(35-84)	-	101
Gopalkrishna V; 1995	10	-	-	0	10	-	-	-	-	10
Gould VE; 2010	33	23	10	8	15	-	-	59 (17-75)	-	33
Helal Tel A; 2006	114	-	-	12	102	-	-	50.8 (8.32/36-69)	-	114
Javanmard B; 2019	110	-	-	14	96	-	-	61.6 (10/32-85)	-	110
Kamel D; 1995	47	-	-	-	-	-	-	-	-	47
Kim KH; 1995	23	-	-	-	-	-	-	-	-	23
Kim SH; 2014	47	35	12	6	29	4	8	71.2 (7.7)	-	47
LaRue H; 1995	70	-	-	-	-	-	-	-	-	70
Llewellyn MA; 2018	689	-	-	-	-	-	-	-	-	-
Lopez-Beltran A; 1996a	76	-	-	14	62	-	-	66.6 (1.17)	-	76

López-Beltrán A; 1996b	76	-	-	14	62	-	-	66.6 (1.17)	-	76
Mete UK; 2018	60	50	10	2	48	-	-	54.1 (44-79)	60	60
Moghadam SO; 2020	106	-	-	21	85	-	-	62.98 (10.26)	-	106
Musangile FY; 2021	228	162*	-	46	182	-	-	74.8 (9.1)	-	228
Pichler R; 2015	186	-	-	37	149	-	-	72 (24-93)	-	186
Samarska IV; 2019	38	-	-	13	25	-	-	-	-	38
Sarier M; 2019	138	69	69	11	58	10	59	63.2 (12.6)	138	-
Schmid SC; 2015	135	109	26	21	88	0	26	73** (45-94)	135	135
Shaker OG; 2013	95	70	25	-	-	-	-	-	-	95
Shigehara K; 2011	127	117	10	23	94	-	-	68.8 (36-89)	124	124
Shigehara K; 2013	84	-	-	84	-	-	-	68.8 (13.5)	-	84
Simoneau M; 1999	187	187	-	-	-	-	-	-	-	-
Steinestel J; 2013	40	19	21	2	17	18	3	74 (9.93)	-	60
Tekin MI; 1999	52	42	10	-	-	-	-	-	42	-
Tenti P; 1996	79	-	-	10	69	-	-	66.2 (23.01)	-	79
Westenend PJ; 2001	16	-	-	7	9	-	-	70 (54-96)***	-	16
Yan Y; 2021	146	-	-	24	122	-	-	66.64 (10.6)	146	-
Yavuzer D; 2011	70	-	-	9	61	-	-	61.9 (13.4)	-	70
Youshya S; 2005	98	-	-	-	-	-	-	73 (21-95)	20	98

*66 tumor samples out of 228 were not included since were not of primary origin **median ***IQR Age by sex was present in 4/46 studies Age by controls was present in 5/46 studies

Table S3. Summary of histological subtypes and of information on tumour differentiation/grade of lesion.

First author	Histological subtypes n/N (%)			Differentiation grade, n/N (%)			Grade of lesion, n/N (%)	
	SCC	UC	TCC	G1	G2	G3	Low	High
Abdollahzadeh P; 2017	-	-	67/67 (100)	1/67 (1.5)	24/67 (35.8)	67/67 (100)	-	-
Aggarwal S; 2009	-	33/33 (100)	-	-	-	-	20/33 (60.6)	13/33 (39.4)
Alexander RE; 2012	42/69 (60.9)	27/69 (39.1)	-	-	-	-	-	-
Alexander RE; 2013	-	-	-	-	-	-	-	-
Alexander RE; 2014	-	-	-	-	-	-	-	-
Badawi H; 2008	4/60 (6.7)	-	25/60 (41.7)	-	-	-	-	-
Barghi MR; 2005	-	-	59/59 (100)	-	-	-	50/59 (84.7)	9/59 (15.3)
Ben Selma W; 2010	5/125 (4)	119/125 (95.2)	-	37/119 (31.1)	49/119 (41.2)	33/119 (27.7)	-	-
Berrada N; 2013	-	42/48 (87.5)	-	-	-	-	13/40 (32.5)	24/40 (60)
Chan KW; 1997	-	-	20/30 (66.7)	-	-	-	-	-
Chapman-Fredricks JR; 2013	14/14 (100)	-	-	-	-	-	-	-
Collins K; 2020	8/33 (24.2)	25/33 (75.8)	-	-	-	-	-	-
Cooper K; 1997	25/25 (100)	-	-	-	-	-	-	-
De Gaetani C; 1999	-	-	43/43 (100)	3/43 (7.0)	24/43 (55.8)	16/43 (37.2)	-	-
Fioriti D; 2003	-	-	-	-	-	-	-	-
Gazzaniga P; 1998	-	-	35/35 (100)	-	-	-	-	-
Golovina DA; 2016	-	-	101/101 (100)	12/101 (11.9)	48/101 (47.5)	41/101 (40.6)	-	-
Gopalkrishna V; 1995	-	-	10/10 (100)	-	-	-	-	-
Gould VE; 2010	-	-	5/23 (21.7)	-	-	-	-	-
Helal Tel A; 2006	32/114 (28.1)	-	67/114 (58.8)	-	-	-	61/99 (61.6)	38/99 (38.4)
Javanmard B; 2019	-	-	110/110 (100)	-	-	-	60/110 (54.5)	50/110 (45.5)
Kamel D; 1995	7/47 (14.9)	-	40/47 (85.1)	15/47 (31.9)	19/47 (40.4)	13/47 (27.7)		
Kim KH; 1995	-	-	23/23 (100)				6/20 (30)	14/20 (70)
Kim SH; 2014	35/35 (100)	-	-	-	-	-	4/33 (12.1)	29/33 (87.9)
LaRue H; 1995	-	-	70/70 (100)	11/70 (15.7)	31/70 (44.3)	28/70 (40.0)	-	-
Llewellyn MA; 2018	-	-	-	-	-	-	-	-
Lopez-Beltran A; 1996a	-	-	76/76 (100)	14/76 (18.4)	28/76 (36.8)	34/76 (44.7)	-	-

López-Beltrán A; 1996b	-	-	76/76 (100)	14/76 (18.4)	28/76 (36.8)	34/76 (44.7)	-	-
Mete UK; 2018	-	50/50 (100)	-	-	-	-	22/50 (44)	28/50 (56)
Moghadam SO; 2020	-	-	106/106 (100)	-	-	-	59/106 (55.7)	47/106 (44.3)
Musangile FY; 2021	-	162/162 (100)	-	-	-	-	-	-
Pichler R; 2015	-	186/186 (100)	-	75/186 (40.3)	77/186 (41.4)	34/186 (18.3)	119/186 (64)	67/186 (36)
Samarska IV; 2019	38/38 (100)	-	-	-	-	-	-	-
Sarier M; 2019	-	69/69 (100)	-	-	-	-	32/69 (46.4)	37/69 (53.6)
Schmid SC; 2015	-	109/109 (100)	-	41/109 (37.6)	-	68/109 (62.4)	-	-
Shaker OG; 2013	15/70 (21.4)	45/70 (64.3)	-	-	-	-	24/45 (53.3)	21/45 (46.7)
Shigehara K; 2011	4/117 (3.4)	-	106/117 (90.6)	28/117 (23.9)	71/117 (60.7)	18/117 (15.4)	-	-
Shigehara K; 2013	4/84 (4.8)	76/84 (90.5)		16/82 (19.5)	47/82 (57.3)	19/82 (23.2)	5/84 (6)	-
Simoneau M; 1999	-	-	187/187 (100)	41/187 (21.9)	98/187 (52.4)	24/187 (12.8)	-	-
Steinestel J; 2013	-	19/19 (100)	-	-	-	-	-	-
Tekin MI; 1999	-	-	42/42 (100)	16/42 (38.1)	14/42 (33.3)	12/42 (28.6)	-	-
Tenti P; 1996	-	-	79/79 (100)	25/79 (31.7)	22/79 (27.8)	32/79 (40.5)	-	-
Westenend PJ; 2001	16/16 (100)	-	-	-	-	-	-	-
Yan Y; 2021	-	-	-	-	-	-	36/127 (28.3)	91/127 (71.7)
Yavuzer D; 2011	-	70/70 (100)	-	-	-	40/70 (57.1)	30/70 (42.9)	40/70 (57.1)
Youshya S; 2005	-	-	98/98 (100)	-	-	-	-	-

Table S4. HPV detection method employed in the selected studies. 1 and 0 indicate that the methodology was adopted, or not, respectively.

First author	Detection method			First author	Detection method		
	PCR	IHC	ISH		PCR	IHC	ISH
Abdollahzadeh P; 2017	0	1	0	*LaRue H; 1995*	1	0	0
Aggarwal S; 2009	1	0	0	*Llewellyn MA; 2018*	1	0	0
Alexander RE; 2012	0	1	1	*Lopez-Beltran A; 1996a*	1	0	0
Alexander RE; 2013	0	1	1	*López-Beltrán A; 1996b*	1	1	1
Alexander RE; 2014	0	1	1	*Mete UK; 2018*	1	0	0
Badawi H; 2008	1	0	0	*Moghadam SO; 2020*	1	0	1
Barghi MR; 2005	1	0	0	*Musangile FY; 2021*	1	1	0
Ben Selma W; 2010	1	0	0	*Pichler R; 2015*	1	0	0
Berrada N; 2013	1	0	0	*Samarska IV; 2019*	1	0	1
Chan KW; 1997	1	1	0	*Sarier M; 2019*	1	0	0
Chapman-Fredricks JR; 2013	1	1	1	*Schmid SC; 2015*	1	0	0
Collins K; 2020	0	1	1	*Shaker OG; 2013*	0	0	1
Cooper K; 1997	1	0	1	*Shigehara K; 2011*	1	1	1
De Gaetani C; 1999	0	0	1	*Shigehara K; 2013*	1	1	1
Fioriti D; 2003	1	0	0	*Simoneau M; 1999*	1	0	0
Gazzaniga P; 1998	1	0	0	*Steinestel J; 2013*	1	0	0
Golovina DA; 2016	1	0	0	*Tekin MI; 1999*	1	0	0
Gopalkrishna V; 1995	1	0	1	*Tenti P; 1996*	1	1	0
Gould VE; 2010	1	1	0	*Westenend PJ; 2001*	0	0	1
Helal Tel A; 2006	1	1	1	*Yan Y; 2021*	1	0	0
Javanmard B; 2019	1	0	0	*Yavuzer D; 2011*	1	0	0
Kamel D; 1995	1	1	1	*Youshya S; 2005*	1	1	0
Kim KH; 1995	1	0	0	**Total %**	**PCR**	**IHC**	**ISH**
Kim SH; 2014	1	1	0		80.4	34.8	37

Table S5: HPV prevalence by genotypes. Prevalence of low risk (LR) HPV6 and HPV11, of high risk (HR) HPV16 and HPV18, and of other HR genotypes was calculated on the total number of identified genotypes. Other HR includes the following genotypes: HPV31, HPV33, HPV35, HPV39, and HPV52.

**First author's name**	**Prevalence by HPV genotypes**				
	HPV6	HPV11	HPV16	HPV18	Other HR
Abdollahzadeh P; 2017	-	-	-	-	-
Aggarwal S; 2009	-	-	4/18 (22.2)	4/18 (22.2)	-
Alexander RE; 2012	-	-	-	-	-
Alexander RE; 2013	-	-	-	-	-
Alexander RE; 2014	-	-	-	-	-
Badawi H; 2008	-	-	5/21 (23.8)	5/21 (23.8)	-
Barghi MR; 2005	4/24 (16.7)	-	17/24 (70.8)	17/24 (70.8)	3/24 (12.5)
Ben Selma W; 2010	-	-	-	-	-
Berrada N; 2013	-	-	-	-	2/27 (7.4)
Chan KW; 1997	-	-	6/7 (85.7)	6/7 (85.7)	-
Chapman-Fredricks JR; 2013	-	-	-	-	1/2 (33.3)
Collins K; 2020	-	-	-	-	-
Cooper K; 1997	-	-	-	-	-
De Gaetani C; 1999	-	-	-	-	-
Fioriti D; 2003	1/1 (100)	-	-	-	-
Gazzaniga P; 1998	-	-	5/11 (45.5)	5/11 (45.5)	
Golovina DA; 2016	-	-	-	-	-
Gopalkrishna V; 1995	-	-	-	-	-
Gould VE; 2010	-	1/7 (14.3)	1/7 (14.3)	1/7 (14.3)	-
Helal Tel A; 2006	-	-	-	-	-
Javanmard B; 2019	-	-	27/52 (24.5)	27/52 (24.5)	-
Kamel D; 1995	13/81 (48.1)	10/81 (37)	16/81 (59.3)	16/81 (59.3)	-
Kim KH; 1995	-	-	8/12 (100)	8/12 (100)	-
Kim SH; 2014	-	-	5/6 (83.3)	5/6 (83.3)	1/6 (16.7)
LaRue H; 1995	-	-	-	-	-

Llewellyn MA; 2018	-	-	-	-	-
Lopez-Beltran A; 1996	-	-	-	-	-
López-Beltrán A; 1996	-	-			
Mete UK; 2018	-	-	-	-	-
Moghadam SO; 2020	1/23 (4.2)	6/23 (25)	6/23 (25)	6/23 (25)	-
Musangile FY; 2021	-	-	-	-	-
Pichler R; 2015	2/3 (50)	-	-	-	-
Samarska IV; 2019	-	-	-	-	-
Sarier M; 2019	-	-	-	-	-
Schmid SC; 2015	-	-	-	-	-
Shaker OG; 2013	-	-	-	-	-
Shigehara K; 2011	-	-	4/15 (22.2)	4/15 (22.2)	8/15 (44.4)
Shigehara K; 2013	1/5 (20)	-	-	-	1/5 (20)
Simoneau M; 1999	3/14 (18.8)	-	3/14 (18.8)	3/14 (18.8)	-
Steinestel J; 2013	-	-	-	-	-
Tekin MI; 1999	-	-	-	-	1/1 (50)
Tenti P; 1996	-	-	10/33 (30.3)	10/33 (30.3)	-
Westenend PJ; 2001	-	-	-	-	-
Yan Y; 2021	-	-	36/64 (85.7)	36/64 (85.7)	19/64 (29.7)
Yavuzer D; 2011	-	-	-	-	-
Youshya S; 2005	-	-	-	-	-

Figure S1. Pooled risk difference by gender [20, 22, 24-25, 29-30, 33, 37, 40-43, 45-46, 51, 57].

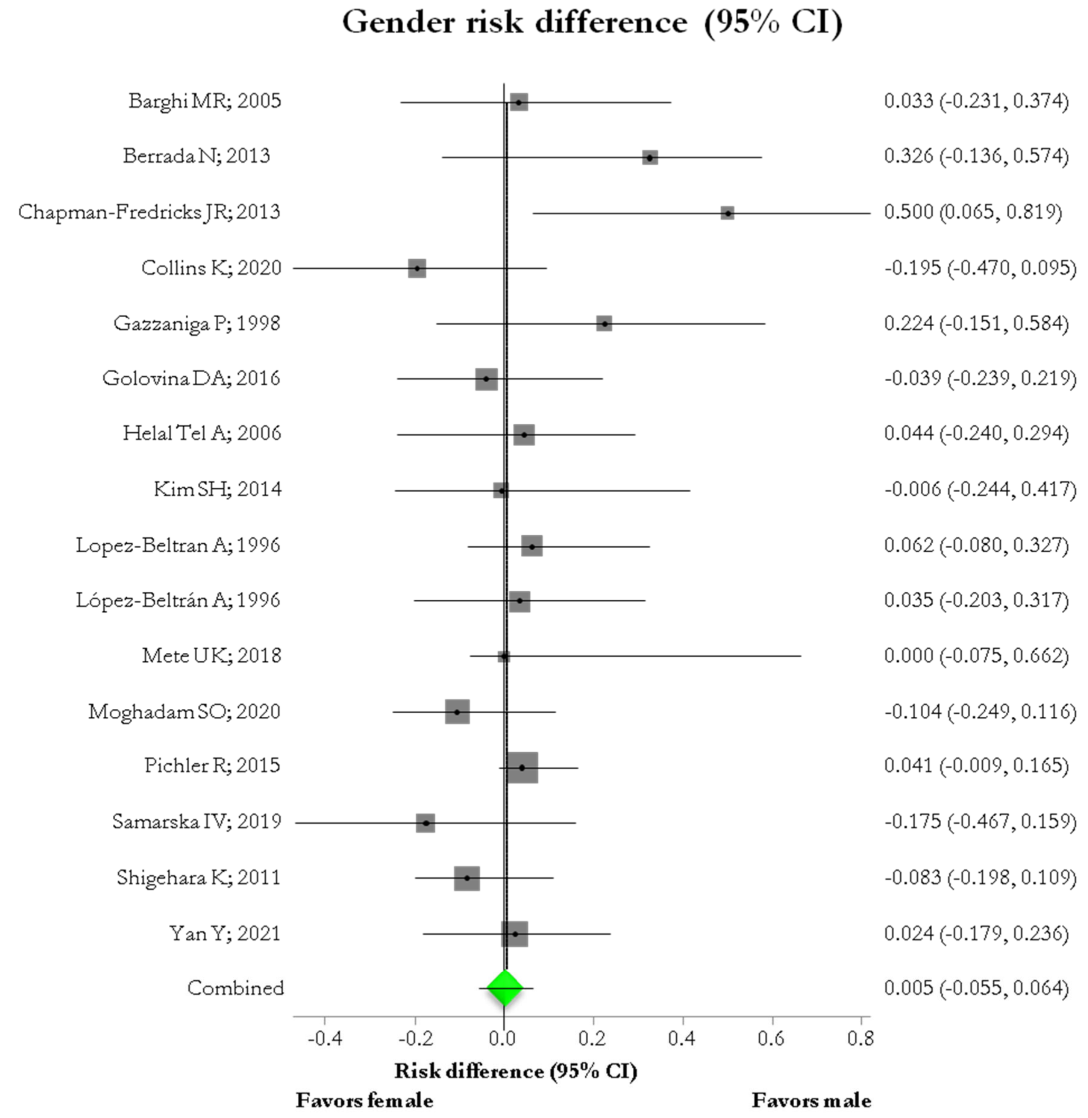

Fixed effects

Pooled risk difference (95% CI) = 0.0046 (-0.0545; 0.0636)

Chi2 (test risk difference differs from 0) = 0.0228 (df = 1) p-value = 0.87999

I^2 (95% CI) = 13.5% (0% - 52.5%)

Figure S2. Pooled risk difference by tumor grading [14, 27, 30, 35-36, 38, 40-41, 45, 50-52, 54].

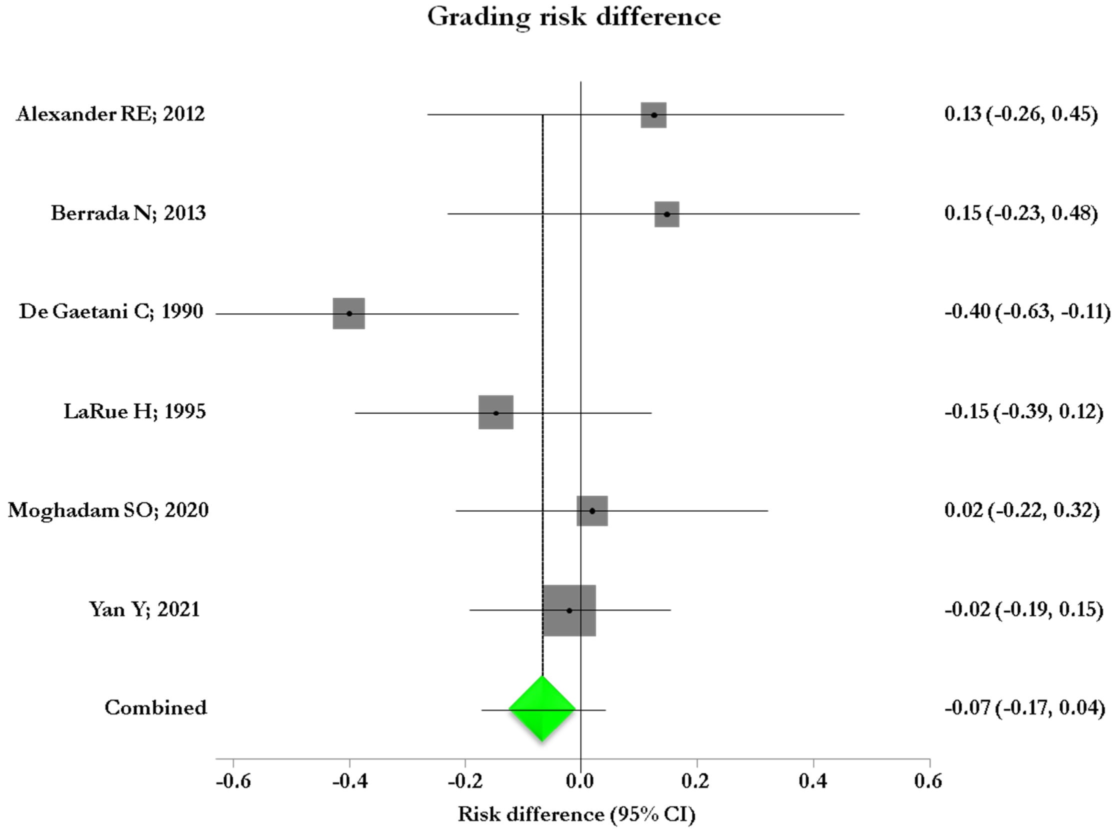

Random effects
Pooled risk difference (95% CI) = -0.0659 (-0.173; 0.0411)
Chi2 (test risk difference differs from 0) = 1.4567 (df = 1) p-value = 0.22746
I^2 (95% CI) = 45.2% (0%-76.7%)

Figure S3. Pooled risk difference by tumor stage [16, 22, 27, 38, 43, 57].

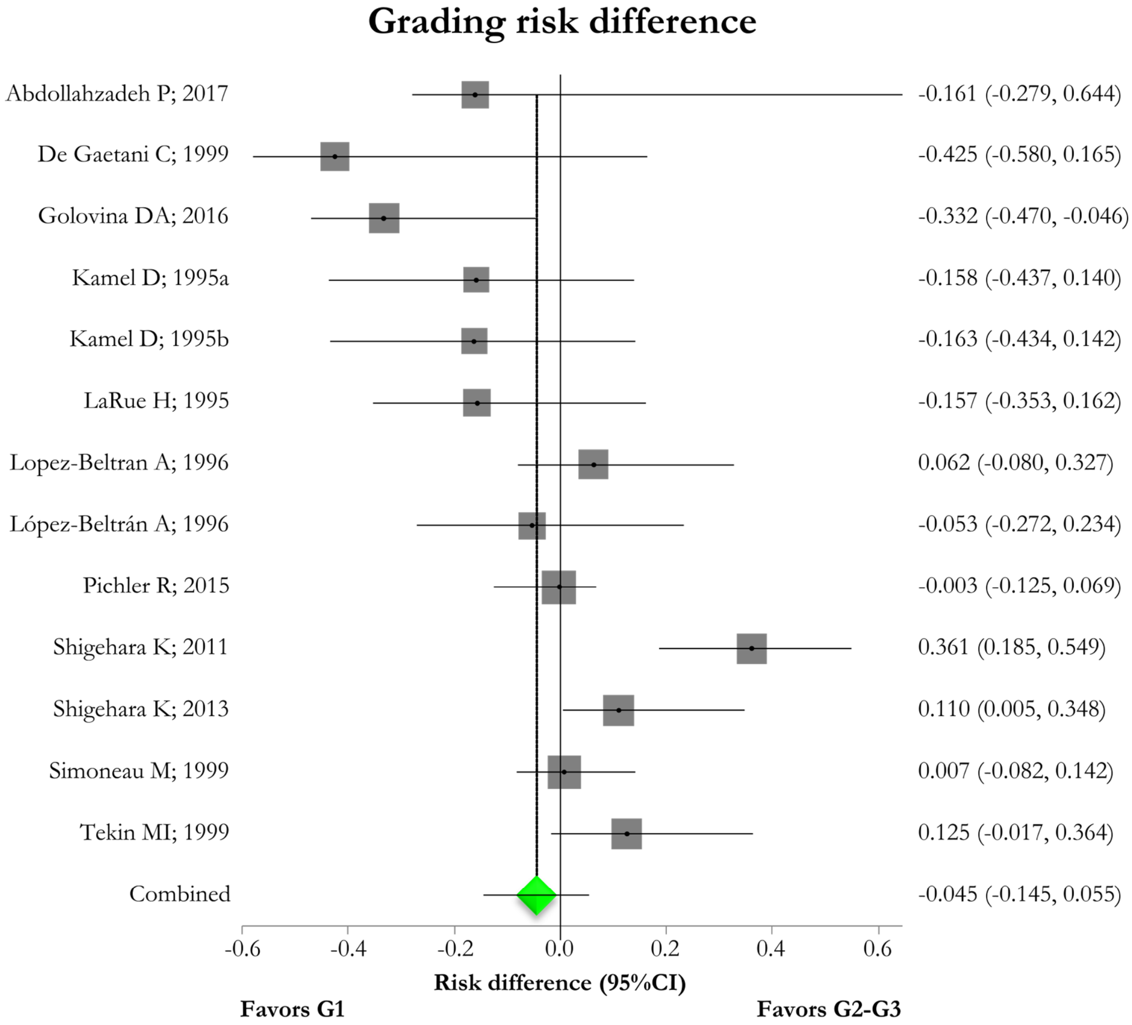

Random effects

Pooled risk difference (95% CI) = -0.0451 (-0.1447; 0.0546)

Chi2 (test risk difference differs from 0) = 0.7857 (df = 1) p-value = 0.37542

I^2 (95% CI) = 75.9% (55%-84.7%)
